# Short-course neoadjuvant chemoradiotherapy and surgery are beneficial in Chinese patients: A retrospective study

**DOI:** 10.1097/MD.0000000000009394

**Published:** 2017-12-22

**Authors:** Ming Jun Huang, Xiao Dong Wang, Yan Jie Hu, Jie Yang, Ka Li

**Affiliations:** a Nursing Department; b Department of Gastrointestinal Surgery; c Department of Hepatic Surgery, West China Hospital, Sichuan University, Chengdu, Sichuan, China.

**Keywords:** neoadjuvant chemoradiotherapy, rectal cancer, short-course

## Abstract

Preoperative neoadjuvant chemoradiotherapy (NACR) is used to reduce tumor size for easier resection or improved resectability rates. Considering the difficulties regarding health insurance and health resources in China, an evidence-based short-course neoadjuvant chemoradiotherapy with surgery to cure patients was performed. This study compared the postoperative effects between short-course neoadjuvant chemoradiotherapy and surgery and surgery without neoadjuvant chemoradiotherapy.

The current retrospective study was based on a rectal cancer database, including 274 patients diagnosed with rectal cancer between January 2014 and October 2016. Data were analyzed with respect to curative rate, postoperative recovery indicators (times to nasogastric tube, urinary catheter, and drainage tube removal and times to first oral feeding and passing of flatus postsurgery), chemoradiotherapy-related indicators [white blood cell count (WBC) and carcinoembryonic antigen (CEA) levels], and adverse effects indicators, evaluated according to Common Terminology Criteria for Adverse Events Version 4.0.

There was no significant difference between the combined therapy and surgery groups (*P* > .05) in terms of radical resection rates and the times to urinary catheter removal and passing flatus (*P* > .05). Statistically significant differences (*P* < .05) in terms of earlier time for removal of the nasogastric and drainage tubes and time to first oral feeding were observed in the combined therapy group. The decreases in WBC and CEA levels in the combined therapy group were significantly greater than those in the surgery group 1 week after surgery (*P* < .05); after 1 month, the CEA decrease in the combined therapy group was significantly greater than that in the surgery group (*P *< .05). More patients in the combined therapy group experienced vomiting, indigestion, dehydration, oral mucositis, sensory neuritis, and alopecia compared with those in the surgery group 1 week after surgery (*P* < .05); after 1 month, only the incidence of alopecia was higher in the combined therapy group (*P* < .05).

The combined therapy group demonstrated earlier postoperative recovery compared with the surgery group. Short-course neoadjuvant chemoradiotherapy with surgery may lead to postoperative treatment-related adverse effects of varying degrees; however, these adverse effects eventually improve with time.

## Abstract 2

Supplemental Digital Content is available in the text

## 1 Introduction

Rectal cancer (RC) remains one of the most common and dangerous gastrointestinal malignant tumors, with a significant economic and health burden in China because of its increasing incidence.^[1–3]^ Although surgery remains the primary treatment for RC, the National Comprehensive Cancer Network (NCCN) Guidelines (2012)^[4–5]^ recommend neoadjuvant chemoradiotherapy before surgery in high-risk patients identified as having Stage II or Stage III-IV disease during preoperative assessment. Clinical evidence suggests that neoadjuvant chemoradiotherapy before surgery could effectively reduce the tumor size, which would debase the disease staging as well make resection easier or improve the resectability rate.^[6–10]^

The NCCN (2012) guidelines^[4]^ recommend neoadjuvant chemoradiotherapy as a long-course treatment consisting of chemotherapy biweekly for at least 2 to 3 months and radiotherapy with a total dose of 45–50 Gy spanning at least 5 weeks. This recommendation presents unique challenges in China, where most patients have to pay for their treatments as there is limited coverage from public or private health insurance.^[11–12]^ This is further compounded by the uneven health resource distribution between urban and rural areas, as it is common for only large hospitals located in cities to offer high-quality medical care, including chemoradiotherapy. Thus, patients in the low economic strata and rural regions have difficulty completing the recommended NACR, prompting physicians in China to launch a modified regimen shortening the NACR to 5 days to achieve the best benefits for patients with limited resources.^[13]^

However, the advantages of short-course neoadjuvant chemoradiotherapy, including shorter treatment times, lower cost, and weaker drug adverse effects, less reportedly impact the quality of patients’ lives.^[14–16]^ Moreover, no significant statistical difference was reported in local remission rate and long-course survival benefits between long-course and short-course neoadjuvant chemoradiotherapy,^[14–16]^ even though the rate of adverse events in long-course chemoradiotherapy were higher than those of the short course in RC patients.^[17]^ As a result, the National Institute for Health and Clinical Excellence (NICE) guidelines in England also recommended short-course NACR in patients with locally advanced RC.^[18]^

Adding steps in the new treatment method inevitably leads to additional risks of side effects related to surgery, postoperative recovery, and relative nursing interventions.^[19]^ For these patients, professional nurses involved in colorectal surgery need to be aware of the potential clinical problems and implement corresponding management approaches. This study evaluated the impact of short-course neoadjuvant chemoradiotherapy on postoperative recovery in our institution. A clinical team in our colorectal surgery department performed a retrospective controlled study to compare the postoperative effects between short-course NACR and standard surgery without NACR.

## 2 Materials and methods

### 2.1 Patients

A total of 274 patients diagnosed with rectal cancer were selected for this retrospective review. These patients were diagnosed in our department between January 2014 and October 2016; the surgery group was enrolled between January 2014 and January 2015, whereas the combined therapy (short-course neoadjuvant chemoradiotherapy and surgery) group was enrolled between October 2015 and October 2016 based on the following criteria (Chart 1):

a. Consented to surgery.
b. Diagnosed with colorectal cancer by preoperative colonoscopy and postoperative pathological examination.
c. Initial diagnosis of colorectal cancer.
d. TNM (tumor, node, and metastasis) stage I or IV cases were excluded.
e. Patients that experienced morbidity such skin changes [eg, eczema, urticaria, neurodermatitis, and systemic lupus erythematosus (SLE) or immune dysfunction (eg, human immunodeficiency virus (HIV), aplastic anemia, and leukemia] during chemotherapy or radiotherapy prior to this hospitalization were excluded.
f. Patients who received chemotherapy or radiotherapy 1 month after surgery and those who were taking medications with potential adverse effects similar to those of chemotherapeutic agents, including hormones and immunosuppressive drugs, were also excluded.
g. We also excluded patients with more than 20% missing data.

Of 198 cases available after screening, 136 were male and 62 were female. Based on the pathologic diagnoses, adenocarcinoma comprised 187 of the cases; the remaining 11 cases were mucinous adenocarcinoma type. Thirty-three cases presented with a well-differentiated glandular formation, 72 were moderately differentiated, and 93 were poorly-differentiated tumors. Using the classification criteria of the American Joint Committee on Cancer (AJCC) and the International Union Against Cancer (UICC),^[20]^ 94 and 104 cases were categorized as Stage II and Stage III disease, respectively. Patient ages ranged from 28 to 80 years and the mean age was 62.6 years. The patients were divided to the combined therapy (82 cases) and surgery (116 cases) groups. Both groups had the same baseline data (Table 1). The study received ethical approval from the clinical research ethics committees (2012–267) of West China Hospital of Sichuan University.

**Table 1:**
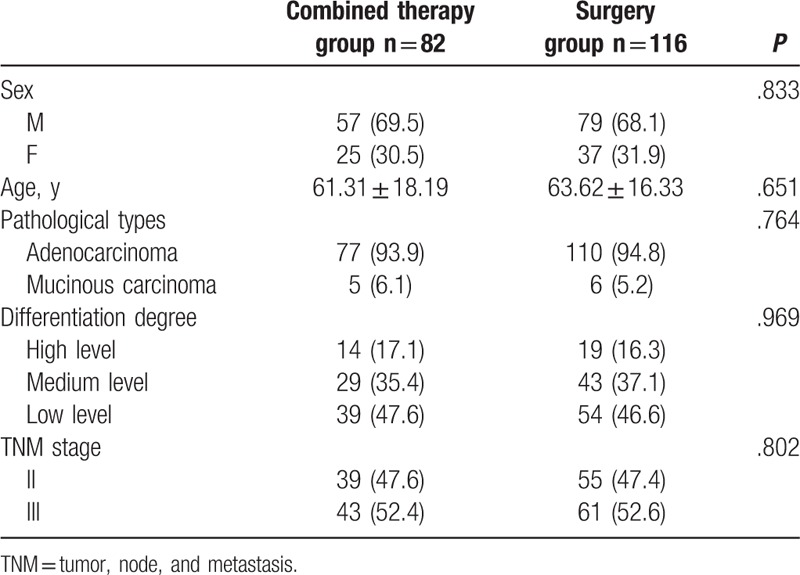
The comparison of basic data between Combined Therapy group and Surgery group (n = 198).

### 2.2 Data collection

The collected data included hospital admission and 1-month postsurgery clinical follow-up records obtained from the Medical Records department. Our department has a professional staff that liaises with patient groups by regularly conducting telephone follow-ups. The periods included in the study were from admission and 1 month postsurgery, whereas the observation points were before neoadjuvant chemoradiotherapy and 1 week and 1 month after surgery. Data were saved in Excel (Office 2007, Microsoft Corp., Seattle, WA).

### 2.3 Develop groups

Patients in the combined therapy group consented to under neoadjuvant chemoradiotherapy and subsequent operation after 1 week. This plan included 5 × 5 Gy radiotherapy for 5 consecutive days with a concurrent cycle of FOLFOX at day 2 of radiotherapy. The FOLFOX regimen included oxaliplatin 85 mg/m^2^ (ivgtt, 2 hrs) + LV 400 mg/m^2^ (ivgtt, 2 hrs) + 5-FU 400 mg/m^2^ (iv.) and began to receive 5-FU 1200 mg/m^2^/d (consecutive ivgtt, 46–48 hrs).

Patients in the surgery group received only surgery, which was a laparotomy procedure, including corrective operation and a palliative procedure (treatments not performed to resect or debulk the tumor).

### 2.4 Evaluation indicators

We evaluated the effect of neoadjuvant chemoradiotherapy on treatment and patient recovery by comparing curative rates. The curative rate was defined as the number of radical resection cases divided by the total number of cases, multiplied by 100%. The postoperative recovery indicators include the times to remove the nasogastric tube, urinary catheter, and drainage tube and the times to first oral feeding and first passing of flatus postsurgery. The chemoradiotherapy-related indicators included white blood cell (WBC) count and carcinoembryonic antigen (CEA) level. We compared the last available data before the administration of NACR to the values measured 1 week or 1 month after surgery. The data values before NACR were subtracted from the postsurgery measurements. The adverse effects indicators were developed according to Common Terminology Criteria for Adverse Events Version 4.0 (CTCAE v4.0)^[21]^ and included:

1. Gastrointestinal reactions: nausea (gastric disorder or urgent need to vomit, lack of appetite), vomiting, indigestion (gastric pain, burning sensation, flatulence, nausea, and vomiting), diarrhea, and gastroesophageal reflux (heartburn and indigestion).
2. Rectum-related phenomena: intestinal obstruction, bleeding in the enteric cavity, intestinal ulceration/perforation, and intestinal stenosis.
3. Fever, oral mucositis, acne-like rashes, sensory neuritis, and alopecia.

### 2.5 Bias control

We categorized the adverse effects into 5 degrees according to CTCAE v4.0: first-degree (low level) was defined as zero or a few symptoms diagnosed in the clinic and that required no intervention. Second-degree (medium level) were defined as diseases requiring local, small, noninvasive therapy or in which the instrumental activities of daily living according to age were limited. Third-degree was defined as the presence of indicators that could lead to hospitalization, lengthen the recovery time, or result in disabilities that were serious or had clinically significant impacts that did not threaten life or limit the personal activities of daily living. Fourth-degree was defined as life-threatening adverse effects that required emergency treatment. Finally, fifth-degree adverse events were defined as death related to adverse effects.

### 2.6 Statistical methods

To control for bias, we optimized the schedule before starting the study and screened clinical cases and follow-up data in the light of the study inclusion and exclusion criteria. Two people logged data and professional statisticians analyzed data; all were blinded during the logging and analysis.

The data were processed using SPSS Statistics for Windows, version 17.0 (SPSS Inc., Chicago, IL). Consecutive variable data with equal variance were assessed using *t* tests; otherwise, *U* tests were used instead. *χ*^*2*^ or Fisher exact tests were used for enumeration data, while Wilcoxon rank-sum tests were used for ranked data based on an α = .05.

## 3 Results

Based on analysis of the curative rates, there was no significant difference between the combined therapy and surgery (*P* > .05) groups in terms of radical resection rates. Among postoperative indicators, there were also no significant differences between groups in the times to urinary catheter removal and passing flatus (*P* > .05). A statistically significant difference (*P* < .05) was observed in the earlier removal times for nasogastric and drainage tubes and the time to first oral feeding in the combined therapy group (Table 2).

**Table 2:**
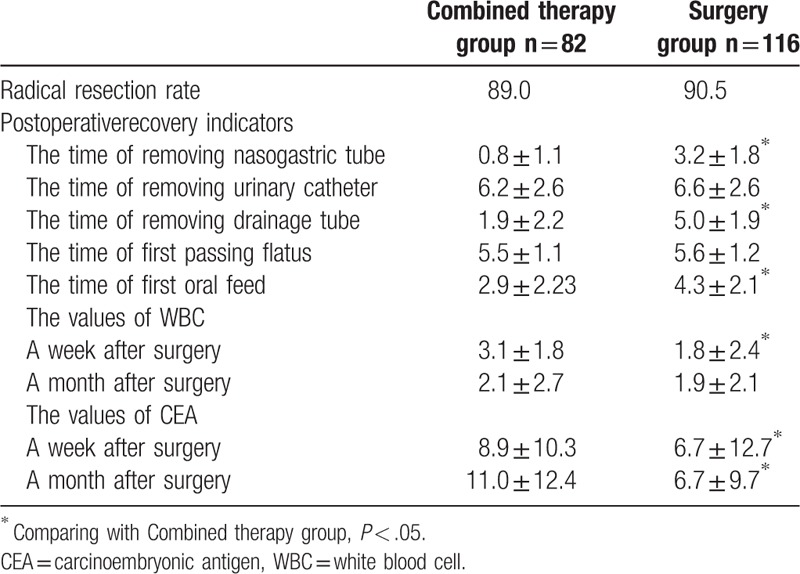
Comparison of recovery a week and a month after surgery between 2 groups.

Analysis of chemoradiotherapy-related indicators 1 week after surgery revealed WBC values of (5.8 ± 2.1) × 10^9^/L and (6.1 ± 2.5) × 10^9^/L before neoadjuvant chemoradiotherapy and (3.0 ± 2.8) × 10^9^/L and (4.4 ± 3.9) × 10^9^/L in the combined therapy and surgery groups, respectively, 1 week after surgery. The WBC decrease was significantly higher in the combined therapy group than that in the surgery group (*P* < .05). The CEA values of patients in the combined therapy and surgery groups were 21.4 ± 25.1 and 26.5 ± 30.7 ng/mL before NACR and 13.8 ± 18.3 and 18.8 ± 21.0 ng/mL, respectively, 1 week after surgery. The decrease in CEA value in the combined therapy group was significantly higher than that in the surgery group (*P* < .05) (Table 2).

No patients experienced gastroesophageal reflux, intestinal perforation, intestinal ulceration, dehydration, or intestinal stenosis one week after surgery. The reported complications, including nausea, diarrhea, intestinal obstruction, hematochezia, fever, and acne-like rashes, did not differ significantly between the groups (*P* > .05). More patients in the combined therapy group experienced vomiting, indigestion, dehydration, oral mucositis, sensory neuritis, and alopecia compared with those in the surgery group (*P* < .05) (Table 3).

**Table 3:**
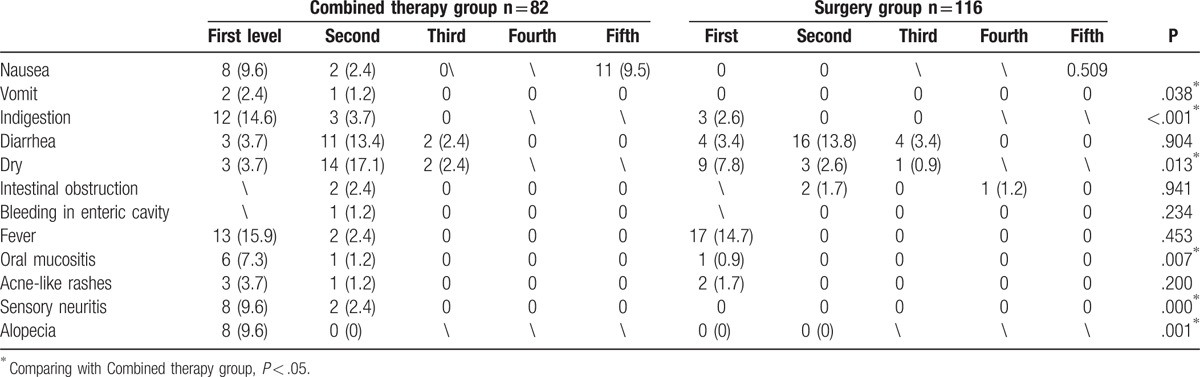
Comparison of adverse effects a week after surgery between 2 groups (n,%).

Assessment of the chemoradiotherapy-related indicators 1 month after surgery revealed WBC in the combined therapy and surgery groups, respectively, of 5.8 ± 2.1 × 10^9^/L and 6.1 ± 2.5 × 10^9^/L before neoadjuvant chemoradiotherapy and 4.8 ± 3.5 × 10^9^/L and 4.7 ± 3.9 × 10^9^/L at 1 month after surgery. The difference in WBC decrease between the groups was not statistically significant (*P* > .05). The CEA values in the combined therapy and surgery groups were 21.4 ± 25.1 and 26.5 ± 30.7 ng/mL before neoadjuvant chemoradiotherapy and 9.5 ± 9.3 and 19.0 ± 20.7 ng/mL 1 month after surgery. The decrease in CEA value in the combined therapy was significantly higher than that in the surgery group (*P* < .05) (Table 3).

No patients in either group experienced complications such as nausea, vomiting, intestinal obstruction, hematochezia, intestinal perforation, intestinal ulceration, fever, acne-like rashes, or oral mucositis 1 month after surgery. The complications included indigestion, diarrhea, gastroesophageal reflux, intestinal stenosis, and sensory neuritis but there were no statistically significant differences between the groups (*P* > .05). Alopecia was significantly more common in the combined therapy group compared with the surgery group (*P* < .05) (Table 4).

**Table 4:**
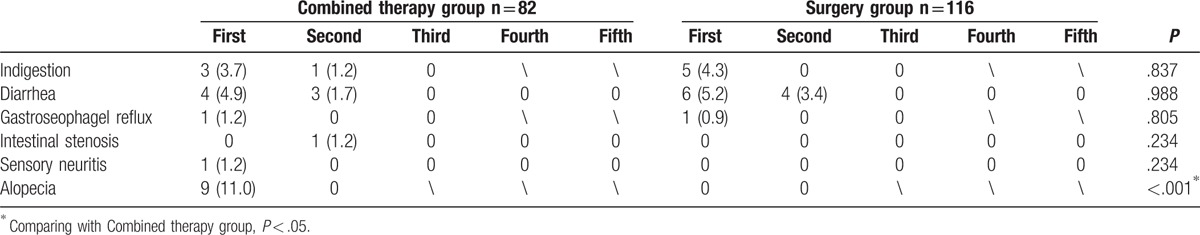
Comparison of adverse effects in a month after surgery between 2 groups (n,%).

## 4 Discussion

The combination of neoadjuvant chemoradiotherapy and surgery is widely acknowledged in international guidelines as a regimen for colorectal cancer treatment.^[4–5]^ There is mounting evidence of the advantages of preoperative neoadjuvant chemoradiotherapy from a number of randomized controlled trials,^[7,22–28]^ leading colorectal surgeons, including those in China, to adopt the practice. A number of neoadjuvant chemoradiotherapy treatment practices were developed in large domestic hospitals, offering patients the benefits described in international scientific papers.^[29–33]^ Easing the economic burden for patients requiring combined therapy was the most crucial reason that this study was undertaken. The combined therapy group in our study demonstrated earlier postoperative recovery than that in the surgery group, thus proving its advantage. Excellent postoperative recovery depends on the reduction in cancer size because of neoadjuvant chemoradiotherapy, which may minimize the surgical risk and difficulty, shorten surgical time, and reduce damage caused by the operation.^[6–10]^ Although the retrospective data did not include the tumor stage and size before surgery, we hope these data will be included as a research point in future prospective studies.

Short-course neoadjuvant chemoradiotherapy may lead to late adverse effects after surgery. Clinical staffs were concerned that the adverse effects caused by the combined therapy would limit satisfaction with hospitalization and compliance with long-course treatment. Neoadjuvant chemoradiotherapy may lead to immune dysfunction, nausea, vomiting, rashes, alopecia, neurodermatitis, and hepatotoxicity.^[34–36]^ It is thus imperative to be aware of adverse event to be prepared to provide an optimal response during the perioperative period to enhance the comprehensive quality when implementing a colorectal treatment plan based on neoadjuvant chemoradiotherapy. Acute adverse effects (eg, nausea and vomiting) are observed in the first few hours after chemoradiotherapy, whereas delayed adverse effects (eg, leukocyte decrease, alopecia, chronic diarrhea, and peripheral, neuropathy) are seen 7 to 9 days after surgery or even later. This period lasted 1 or 2 months or longer in long-course neoadjuvant chemoradiotherapy ^[37]^; however, the preoperative treatment-related adverse effects caused by long-course neoadjuvant chemoradiotherapy were harder to assess. None have been documented so far. Our study aimed to explore the possibility of regulating the adverse effects of short-course neoadjuvant chemoradiotherapy during the perioperative and late recovery periods. Our research came from a single-treatment group, which utilized a computer algorithm recovery mode of fast-track surgery.^[38–39]^ The combined therapy and surgery groups managed with fast-track surgery both showed good postoperative recovery indicators; however, adverse effects related to radiotherapy were reported in the combined therapy group.

The adverse effects of radiotherapy disappeared over time.^[40–41]^ Changes in WBC made clinical sense as an index to evaluate the adverse effects of vein radiotherapy. Patients who received radiotherapy often had experienced decreased leukocyte counts even though leukocyte crisis was the result of malignant myelosuppression.^[42]^ In our study, the WBC values in the combined therapy group were less than those in the surgery group, suggesting that the effects of combined therapy were worse before neoadjuvant chemoradiotherapy or 1 week after surgery. The WBC did not differ significantly between the combined therapy and surgery groups and the drop in CEA in the combined therapy group was significantly less than that in the surgery group 1 month after surgery. Therefore, patients in the recent recovery period may have low WBC levels, which could be an important factor affecting postoperative recovery. Developing a postsurgery recovery plan and discharging at a reasonable time could help to avoid these risks and guarantee good short-course results for combined therapy of malignant tumor. Close monitoring for leukocyte crisis and promptly administering relevant treatment may reduce the risk of infection in patients after surgery.

Although the combined therapy group had an increased probability of developing adverse effects one week after surgery, specifically adverse effects related to vein radiotherapy, adverse effects such as vomiting, oral mucositis, indigestion, sensory neuritis, and alopecia improved and eventually disappeared over time, with no significant differences observed between the groups after 1 month. However, because of its characteristics, alopecia cannot be corrected during this short period; therefore, patients who consent to this procedure must be made aware of this. In general, the adverse effects experienced by patients were generally mild and disappeared over time. Patients with severe adverse effects (third degree or more) included 2 patients with diarrhea and another 2 patients with dehydration 1 week after surgery. The series of problems may be resolved and improved with long-course postoperative follow-up and treatment compliance of patients if a reasonable postoperative short-course recovery plan was arranged beforehand.

Several limitations of this study should be noted. First, this retrospective study was limited in scope to preliminarily test the hypothesis. Future prospective randomized controlled trials are necessary to confirm these findings. Second, long-course chemoradiotherapy is also administered to rectal cancer patients in this clinic; however, we did not compare patients who had received long-course and short-course courses. Therefore, further research is necessary to compare these treatments. Lastly, the 3 and 5-year survival rates were not recorded.

In summary, combining neoadjuvant chemoradiotherapy with surgery may cause postoperative treatment-related adverse effects to varying degrees, notably leucocyte crisis and peripheral neuritis; however, these adverse effects improved with time postsurgery. Furthermore, these adverse effects did not affect the postoperative recovery time.

## Supplementary Material

Supplemental Digital Content
