# Supplementary material for: Short-course neoadjuvant chemoradiotherapy and surgery are beneficial in Chinese patients: A retrospective study
Source: Medicine (Baltimore). 2017 Dec 22;96(51):e9394. doi: 10.1097/MD.0000000000009394 (PMC5758250; doi:10.1097/MD.0000000000009394)
Supplement: Supplemental Digital Content [file medi-96-e9394-s001.docx]

chart 1：

198 cases by final screening

214 cases by second screening

274 cases of rectal cancer

262 cases by preliminary screening

12 cases were rejected according to inclusion criteria, of which 7 patients didn't go through surgery and 5 patients were not diagnosed with rectal cancer at first time.

48 cases were rejected according to exclusion criteria, of which 1 patient was with HIV,1 patient was with chronic eczema, and 31 patients were marked as staging I. 3 patients who accepted radiotherapy or chemotherapy before this hospitalization and 12 patients who received radiotherapy a month after surgery were also excluded.

16 cases according to rejection criteria, of which 6 cases missed data over 20% and 2 cases missed follow-up data of a week after surgery over 20%

Surgery group

N=116

Combined Therapy group

N=82

Chart1. The included flow chart of clinical data

HIV: human immunodeficiency virus
